# HELLS Reduction Contributes to Compressive Force-Induced Functional Changes in PDLSCs

**DOI:** 10.3390/ijms27104540

**Published:** 2026-05-19

**Authors:** Manqi Wang, Qian Li, Jiaqi Chen, Bing Han, Wei Hu

**Affiliations:** 1 National Engineering Laboratory for Digital and Material Technology of Stomatology, Beijing Key Laboratory of Digital Stomatology, Department of Orthodontics, Peking University School and Hospital of Stomatology, Beijing 100081, Chinaqianli@bjmu.edu.cn (Q.L.);; 2 National Center for Stomatology & National Clinical Research Center for Oral Diseases & National Engineering Research Center of Oral Biomaterials and Digital Medical Devices, Beijing 100081, China; 3 Beijing Key Laboratory of Digital Stomatology & NHC Key Laboratory of Digital Stomatology & NMPA Key Laboratory for Dental Materials, Beijing 100081, China

**Keywords:** HELLS, periodontal ligament stem cells, orthodontic tooth movement, compressive force, osteogenic differentiation

## Abstract

Orthodontic tooth movement (OTM) is driven by force-induced alveolar bone remodeling, yet the molecular mechanisms by which periodontal ligament stem cells (PDLSCs) sense and transduce mechanical signals remain incompletely understood. Here, we identify the epigenetic regulator HELLS as a compressive force-responsive gene and investigate its role as a mechanosensitive mediator in human PDLSCs (hPDLSCs). Compressive force downregulated HELLS expression both in vitro and in a mouse OTM model. Functionally, siRNA-mediated HELLS knockdown impaired osteogenic differentiation, as evidenced by reduced Alizarin Red S staining and alkaline phosphatase activity, and induced global transcriptomic changes indicative of altered mechanotransduction pathways. Moreover, HELLS knockdown increased YAP and RANKL expression and potentiated osteoclast differentiation of co-cultured RAW264.7 cells. Finally, we identified E2F1 as a candidate transcription factor mediating the force-induced downregulation of HELLS. Collectively, these findings establish HELLS as a potential mechano-epigenetic regulator in hPDLSCs, and suggest that its force-induced downregulation may contribute to alveolar bone remodeling during OTM by simultaneously attenuating osteogenesis and enhancing pro-osteoclastogenic signaling via transcriptional reprogramming.

## 1. Introduction

Orthodontic tooth movement (OTM) is a complex biological process that involves the dental pulp, periodontal ligament (PDL), alveolar bone, and gingiva. It is fundamentally characterized by the adaptive remodeling of the PDL and alveolar bone in response to mechanical loading. Specifically, bone resorption predominates on the pressure side, while bone formation occurs on the tension side; these synchronized processes collectively facilitate orthodontic tooth movement [1,2].

Within this intricate network of mechanotransduction and tissue remodeling, periodontal ligament stem cells (PDLSCs) have been identified as pivotal regulators [3]. As a distinct population of mesenchymal stem cells, PDLSCs possess multipotent differentiation potential and robust self-renewal capacity [4]. Mechanical stimuli cause changes in the function and behavior of PDLSCs. Notably, in vitro compressive force reduces the osteogenic differentiation potential of PDLSCs [5,6], while simultaneously promoting the production of pro-inflammatory cytokines; among these, Receptor Activator of Nuclear Factor-κB Ligand (RANKL) plays an important role in modulating osteoclast activity [7]. Consequently, elucidating the molecular mechanisms governing behavioral alterations in PDLSCs is essential for optimizing the rate, efficiency, and biological safety of OTM.

Increasing evidence underscores the pivotal role of epigenetic regulation in mechanotransduction [5,8,9,10,11,12]. Epigenetic mechanisms, including DNA methylation, histone modifications, and chromatin remodeling, dynamically modulate gene expression without altering the primary DNA sequence. Consequently, they govern long-term adaptive cellular responses to sustained mechanical loading. Lymphoid-specific helicase (HELLS, also designated SMARCA6), a key member of the SNF2 family of ATPases, is a crucial chromatin remodeling protein [13,14]. By modulating chromatin accessibility, DNA methylation, and transcriptional activity, HELLS plays an indispensable role in maintaining genomic methylation patterns and regulating stem cell homeostasis, proliferation, and lineage commitment [15,16,17,18,19]. However, the specific roles of HELLS in the orthodontic force-induced microenvironment have not been characterized. In this study, the regulatory functions and molecular mechanisms of HELLS were investigated in PDLSCs during OTM through integrated in vivo and in vitro analyses.

## 2. Results

### 2.1. Compressive Force Decreases HELLS Expression in Human PDLSCs and Mouse Periodontal Ligaments

**Figure 1:**
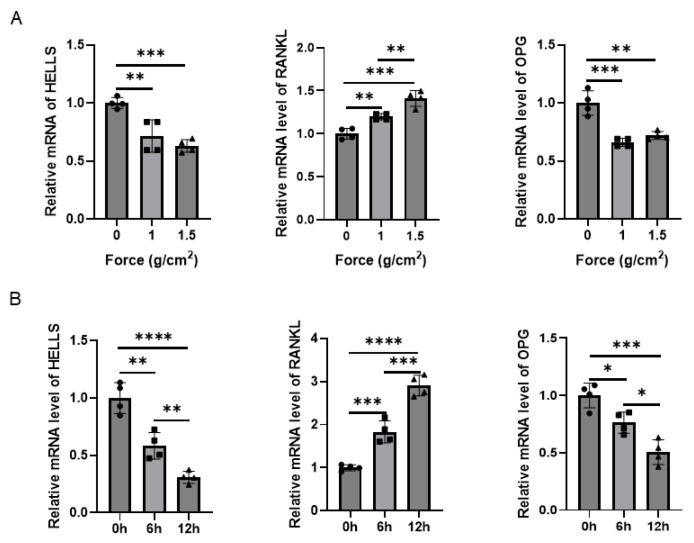
Compressive force decreases mRNA levels of *HELLS* in hPDLSCs. (**A**) qRT-PCR analysis of *HELLS*, *RANKL*, and *OPG* mRNA levels in hPDLSCs subjected to a static compressive force of 0, 1, or 1.5 g/cm^2^ for 6 h. (**B**) qRT-PCR analysis of *HELLS*, *RANKL*, and *OPG* mRNA levels in hPDLSCs under 1 g/cm^2^ compressive force for the indicated durations. Data are presented as means ± SD from three independent experiments (One-way ANOVA; * *p* < 0.05, ** *p* < 0.01, *** *p* < 0.001, **** *p* < 0.0001).

**Figure 2:**
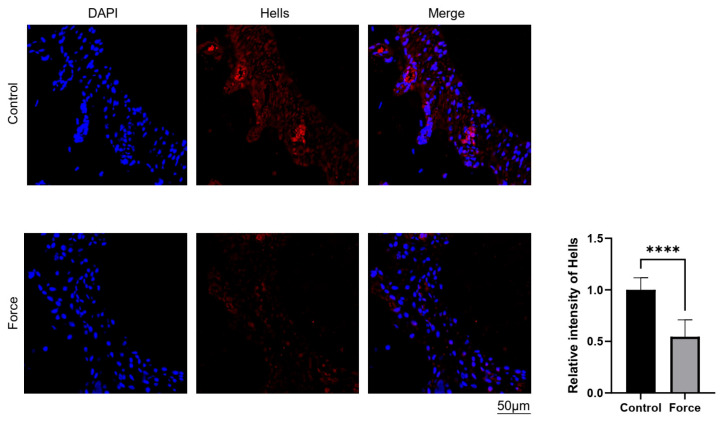
HELLS expression is reduced in the compression-side periodontium during orthodontic tooth movement. Immunofluorescence analysis of HELLS in the periodontal ligament of mice subjected to OTM (orthodontic tooth movement) and control (no force) conditions. DAPI (4′,6-diamidino-2-phenylindole) shows the localization of cellular nuclei. Bar: 50 μm. Data are presented as means ± SD (*n* = 7; Student’s *t*-test, **** *p* < 0.0001).

To identify epigenetic regulators in PDLSCs that respond to mechanical stimuli, we first analyzed RNA sequencing data for PDLSCs subjected to compressive force. Notably, HELLS was significantly downregulated, suggesting that it acts as a mechano-responsive gene involved in modulating PDLSC behavior. To validate this, we performed qRT-PCR to examine expression levels under static compressive force. In human PDLSCs, compressive loading at both 1.0 g/cm^2^ and 1.5 g/cm^2^ for 6 h markedly reduced *HELLS* mRNA levels, although no significant difference was observed between the two force intensities (Figure 1A, left panel). Concurrently, compressive force upregulated *RANKL* and downregulated *OPG* (Figure 1A, right panels), indicating a shift toward a pro-osteoclastic phenotype in PDLSCs. Time-course analysis under a constant load of 1.0 g/cm^2^ revealed a progressive, time-dependent decrease in *HELLS* expression (Figure 1B, left panel). Extended force application also enhanced *RANKL* expression and reduced *OPG* levels in a time-dependent manner (Figure 1B, right panels). Consistent with the in vitro findings, an in vivo mouse OTM model demonstrated that the compression side of periodontal ligament, following 14 days of force application, exhibited a weaker HELLS signal compared with that on the control side (Figure 2). Collectively, these results demonstrate that compressive force reduces HELLS expression in both human PDLSCs and mouse periodontal ligaments.

### 2.2. HELLS Knockdown Impairs the Osteogenic Differentiation Potential of hPDLSCs

**Figure 3:**
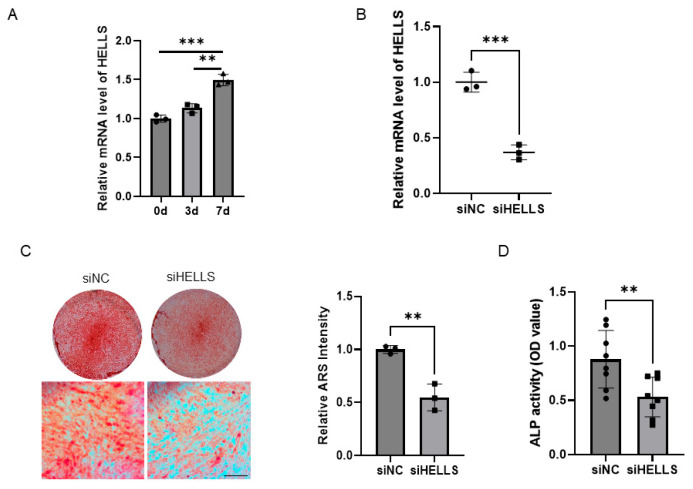
HELLS knockdown impairs osteogenic differentiation of hPDLSCs. (**A**) qRT-PCR analysis of *HELLS* expression during osteogenic induction of hPDLSCs at days 0, 3, and 7 (*n* = 3; one-way ANOVA). (**B**) qRT-PCR confirmation of *HELLS* knockdown in hPDLSCs transfected with siRNA-targeting HELLS (siHELLS) or a negative control siRNA (siNC) (*n* = 3; Student’s *t*-test). (**C**) Alizarin Red staining (ARS) of mineralized matrix deposition in siHELLS- or siNC-transfected hPDLSCs following 14 days of osteogenic induction (bar: 250 μm; *n* = 3; Student’s *t*-test). (**D**) Alkaline phosphatase (ALP) activity in siHELLS- or siNC-transfected hPDLSCs after 14 days of osteogenic induction (*n* = 8; Student’s *t*-test). Data are presented as means ± SD (** *p* < 0.01, *** *p* < 0.001).

To investigate whether HELLS regulates the osteogenic differentiation of PDLSCs, we assessed its expression during osteogenic induction. qRT-PCR analysis revealed a significant increase in *HELLS* mRNA levels over the course of differentiation, suggesting that it contributes to the modulation of the osteogenic capacity of PDLSCs (Figure 3A). We then evaluated the function of HELLS through siRNA-mediated knockdown. Efficient reduction of *HELLS* expression was confirmed by qRT-PCR (Figure 3B). Osteogenic potential was then examined through Alizarin Red Staining (ARS) assay, which exhibited markedly less mineralized nodule formation in HELLS knockdown cells at day 14 compared with that in the controls (Figure 3C). Additionally, Alkaline phosphatase (ALP) enzymatic activity was significantly reduced following HELLS knockdown (Figure 3D). Given that HELLS plays a central role in chromatin remodeling, we examined whether it regulates DNA damage. Immunofluorescence staining of γH2A.X revealed weak signals in both siHELLS- and siNC-transfected PDLSCs, with no apparent difference (Supplementary Figure S2), indicating that HELLS knockdown does not elicit a significant DNA damage response. Taken together, these results indicate that HELLS depletion impairs osteogenic differentiation and matrix mineralization in human PDLSCs.

### 2.3. HELLS Knockdown Induces Significant Transcriptomic Alterations in hPDLSCs

**Figure 4:**
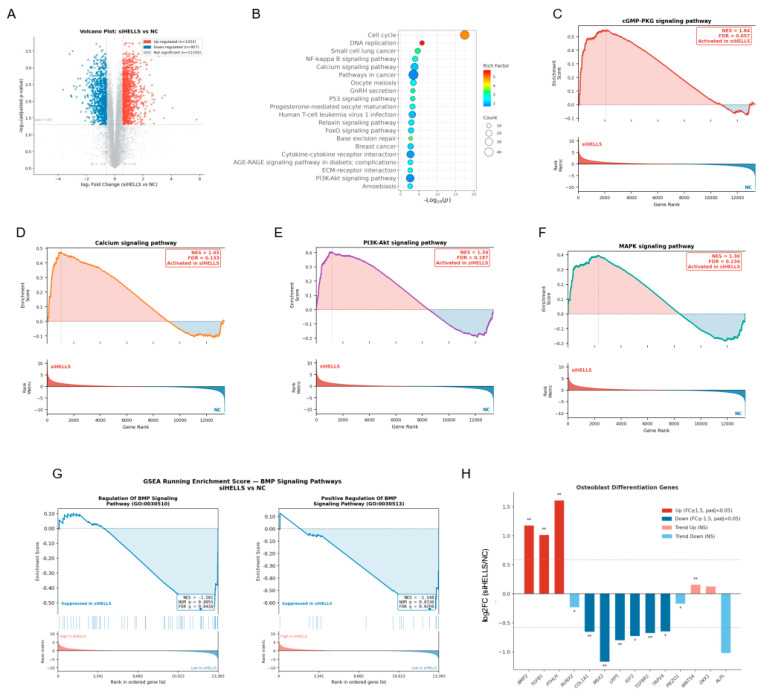
Transcriptomic analysis in HELLS-knockdown and control hPDLSCs. hPDLSCs were transfected with siRNA against HELLS (siHELLS) or a negative control (NC) and then subjected to transcriptomic sequencing. (**A**) The volcano plot of differentially expressed genes (DEGs). (**B**) Kyoto Encyclopedia of Genes and Genomes (KEGG) pathway enrichment analysis of DEGs. (**C**–**F**) Gene-Set Enrichment Analysis (GSEA) running enrichment score plots for four mechanosensing- and bone-related KEGG pathways in siHELLS vs. NC cells. (**G**) GSEA running enrichment score plots for two BMP signaling gene sets in siHELLS vs. NC cells. (**H**) Bar plot showing log_2_ fold change (siHELLS vs. NC) for osteoblast differentiation genes (*n* = 3; Student’s *t*-test; * *p* < 0.05; ** *p* < 0.01).

To identify key molecular pathways regulated by HELLS, we performed transcriptome sequencing on PDLSCs transfected with siHELLS or siNC. Principal component analysis revealed a clear separation between the two groups, thereby indicating substantial global differences in their gene expression profiles (Supplemental Figure S1A). Differential expression analysis identified 2209 significantly altered genes, including 1252 upregulated and 957 downregulated transcripts in PDLSCs transfected with siHELLS (Figure 4A). As expected, *HELLS* expression was reduced to less than 20% of control levels following siRNA knockdown (Supplemental Figure S1B). A Gene Ontology (GO) enrichment analysis demonstrated that the DEGs are predominantly involved in cell cycle-related processes, such as mitotic progression, chromosome segregation, and nuclear division (Supplemental Figure S1C). Concurrently, a Kyoto Encyclopedia of Genes and Genomes (KEGG) pathway analysis highlighted significant enrichment in DNA replication, NF-κB signaling, calcium signaling, and several cancer-associated pathways (Figure 4B). To further explore the impact of HELLS knockdown on mechaotransduction, we performed Gene Set Enrichment Analysis (GSEA). Intriguingly, cGMP-PKG, calcium, PI3K-AKT and MAPK signaling pathway were significantly activated in HELLS-knockdown cells (Figure 4C–F), whereas BMP-related signaling pathways were markedly suppressed (Figure 4G). Additionally, we observed downregulation of multiple genes critical for osteoblast differentiation (Figure 4H). Collectively, these data suggest that HELLS knockdown modulates mechanotransduction in hPDLSCs by orchestrating the coordinated activity of several key signaling pathways and by suppressing the transcriptional program of osteogenic differentiation.

### 2.4. HELLS Depletion Shifts hPDLSCs Toward a Pro-Osteoclastogenic Phenotype

**Figure 5:**
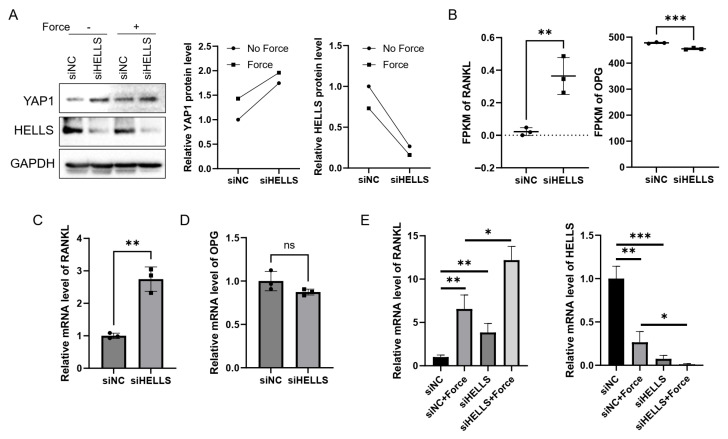
HELLS knockdown upregulates YAP and RANKL in hPDLSCs. (**A**) Western blot analysis of YAP and HELLS protein levels in hPDLSCs transfected with siHELLS or negative control (siNC), with or without static compression (1 g/cm^2^, 12 h). (**B**) RNA-seq expression levels (FPKM, fragments per kilobase per million mapped fragments) of *RANKL* and *OPG* in siHELLS- versus siNC-transfected hPDLSCs. (**C**,**D**) qRT-PCR validation of *RANKL* (**C**) and *OPG* (**D**) expression in siHELLS- and siNC-transfected hPDLSCs (Student’s *t*-test). (**E**) qRT-PCR analysis of *RANKL* and *HELLS* expression in hPDLSCs transfected with siHELLS or siNC, with or without force application (1 g/cm^2^, 12 h). (*n* = 3; One-way ANOVA. * *p* < 0.05; ** *p* < 0.01; *** *p* < 0.001; ns—not significant).

Yes-associated protein (YAP)/ Transcriptional coactivator with PDZ-binding motif (TAZ) was reported to be an important regulator in OTM bone remodeling. We therefore tested whether HELLS knockdown affected YAP expression. Western blot analysis indicated that HELLS silencing upregulates YAP protein levels under both the basal condition and compression application, with the highest YAP expression in cells subjected to both HELLS knockdown and compressive force (Figure 5A). Compressive force is well established to increase the production of RANKL (TNF superfamily member 11), a key cytokine that promotes osteoclast differentiation from mononuclear precursors. This is often accompanied by a decrease in its decoy receptor, OPG (TNF receptor super-family member 11b), under mechanical load [20,21,22]. The resulting shift in the RANKL/OPG ratio is a critical rate-limiting determinant of bone resorption and OTM velocity. Our transcriptomic sequencing data revealed that HELLS knockdown increases *RANKL* mRNA levels substantially and causes a modest decrease in *OPG* expression (Figure 5B). Subsequent qRT-PCR validation confirmed a significant upregulation of *RANKL* in HELLS-deficient cells, while *OPG* exhibited a non-significant decreasing trend (Figure 5C,D). Moreover, HELLS knockdown further upregulates *RANKL* expression compared to force treatment alone, coinciding with the lowest HELLS levels induced by the combination of siRNA and force application (Figure 5E). These findings indicate that force-induced downregulation of HELLS may enhance YAP and RANKL expression, thereby shifting hPDLSCs toward a pro-osteoclastogenic phenotype.

**Figure 6:**
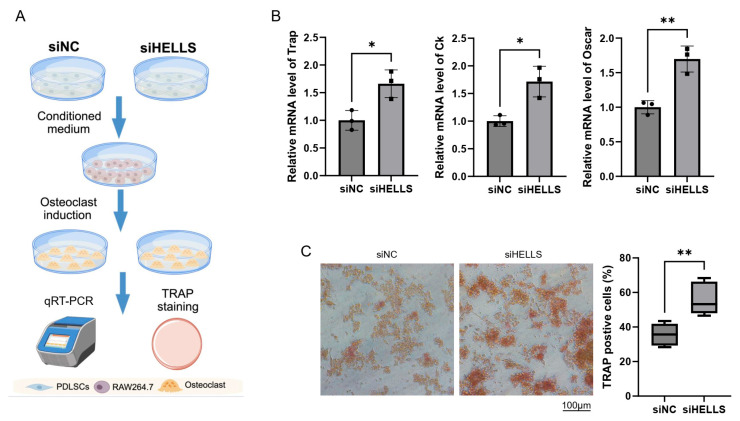
Knockdown of HELLS in hPDLSCs promotes osteoclast differentiation of co-cultured RAW264.7 cells. (**A**) Schematic diagram of the experimental workflow. hPDLSCs were transfected with siHELLS or control siRNA (siNC). The conditioned medium was mixed 1:1 with fresh medium and added to RAW264.7 cells, followed by osteoclast differentiation induction with RANKL (25 ng/mL) for 5 days. Subsequently, qRT-PCR analysis of osteoclast differentiation-associated genes (**B**) and TRAP-staining assay (**C**) were performed (*n* = 3 in (**B**), *n* = 5 in (**C**)). Student’s *t*-test was used for comparisons between siHELLS and siNC groups. * *p* < 0.05, ** *p* < 0.01).

To investigate whether HELLS’ reduction in hPDLSCs affects osteoclast differentiation of mono-macrophages, we cocultured RAW264.7 cells with conditioned media from HELLS-knockdown or control PDLSCs, followed by induction of osteoclast differentiation (Figure 6A). qRT-PCR analysis showed that HELLS knockdown resulted in upregulated expression of osteoclast marker genes, including *Trap*, *Ck*, *and Oscar* in RAW264.7 cells (Figure 6B). Concordantly, Tartrate-Resistant Acid Phosphatase (TRAP) staining revealed enhanced osteoclastogenesis in the HELLS-knockdown group (Figure 6C). These results demonstrate that downregulation of HELLS enhances the pro-osteoclast differentiation capacity of PDLSCs.

### 2.5. E2F1 May Act as a Transcription Factor for HELLS in Response to Compressive Force

**Figure 7:**
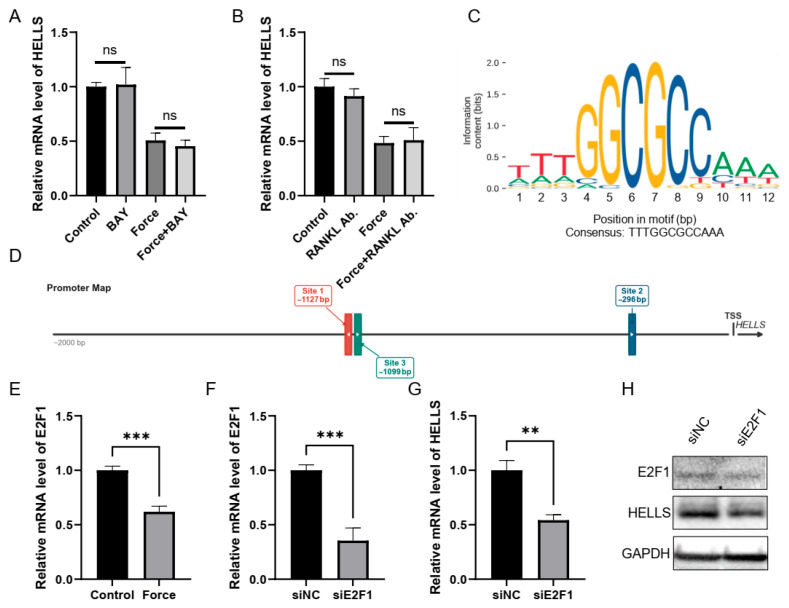
E2F1 is a candidate transcription factor (TF) of HELLS under compressive force. (**A**) qRT-PCR analysis of *HELLS* expression in hPDLSCs with or without administration of BAY 11-7082 (BAY, 5 μM), followed by compressive force treatment (1 g/cm^2^, 12 h) or not. (**B**) qRT-PCR analysis of *HELLS* expression in hPDLSCs with or without administration of RANKL neutralizing antibody (Ab.) followed by compressive force treatment (1 g/cm^2^, 12 h) or not. (**C**) The sequence logo for the E2F1 binding motif was retrieved from JASPAR database. (**D**) Schematic promoter map of the HELLS locus showing the positions of the three predicted E2F1 binding sites (colored boxes) relative to the transcription start site (TSS). (**E**) qRT-PCR analysis of *E2F1* expression in hPDLSCs subjected to compressive force (1 g/cm^2^, 24 h) or control cells. (**F**–**H**) qRT-PCR and western blot analysis for E2F1 and HELLS expression in hPDLSCs transfected with siRNA against E2F1 (siE2F1) or control (siNC) (*n* = 3; Student’s *t*-test; ** *p* < 0.01, *** *p* < 0.001, ns—not significant).

We next sought upstream factors mediating HELLS reduction by compressive force. *HELLS* expression was unaffected by NF-κB inhibitor or RANKL neutralizing antibody, ruling out these pathways (Figure 7A,B). Instead, we focused on E2F1, a mechanosensitive transcription factor (TF) previously linked to the lamin A-RB axis [10]. Given existing reports of E2F1-driven HELLS transcription in cancer cells [23,24], we examined whether E2F1 similarly regulates HELLS under force. JASPAR analysis revealed three E2F1-binding motifs in the HELLS promoter (Figure 7C,D). Compressive force reduced *E2F1* expression in hPDLSCs (qRT-PCR), and E2F1 knockdown suppressed HELLS at both mRNA and protein levels (Figure 7F–H). Collectively, these data identify E2F1 as a candidate TF for HELLS in hPDLSCs under compressive force.

## 3. Discussion

OTM is a biomechanically driven process of periodontal remodeling where hPDLSCs critically balance bone formation and resorption [25]. Understanding how mechanical force directs hPDLSC differentiation is central to orthodontic biology. Here, we characterized the mechanosensitive expression of the chromatin remodeler HELLS, defined its role in regulating hPDLSC osteogenic and osteoclastogenic potential, and mapped its downstream transcriptional landscape.

Our findings identify HELLS as a highly mechanosensitive gene within the periodontal microenvironment, exhibiting a time-dependent decrease under static compression in vitro and diminished expression on the compression side in a mouse OTM model. These results suggest that HELLS functions as a mechano-epigenetic transducer, converting physical stimuli into epigenetic modifications that initiate downstream cellular cascades. Mechanical force is known to modulate chromatin accessibility and epigenetic landscapes [10,26], and our results provide a novel mechanistic link between biomechanical stress and the epigenetic regulation of periodontal gene expression.

Functionally, HELLS is essential for the osteogenic capacity of hPDLSCs, as its knockdown impaired ALP activity and mineralization. Consistently, RNA-seq analysis revealed significant suppression of BMP2 signaling and multiple osteoblast differentiation genes. We propose that HELLS maintains chromatin accessibility at pro-osteogenic loci (e.g., Runx2), thereby facilitating differentiation—an epigenetic mechanism that may explain the inhibited bone formation on the compression side during OTM—though this awaits experimental validation. Furthermore, GSEA identified several mechanosensitive pathways that were markedly suppressed upon HELLS knockdown. HELLS likely fine-tunes the activity of these force-responsive pathways by modulating their chromatin architecture. Future studies are warranted to investigate the precise mechanistic links between HELLS and these critical pathways in OTM.

Several limitations should be acknowledged. First, whether HELLS downregulation is required for force-induced mechanotransduction and functional changes needs to be validated by HELLS overexpression under force loading. Second, chromatin accessibility assays (e.g., ATAC-seq or MNase-seq) are needed to clarify the epigenetic mechanism by which HELLS regulates osteogenic and osteoclast differentiation. Third, the proposed E2F1-mediated transcriptional regulation of HELLS requires experimental confirmation (e.g., ChIP assay and E2F1 overexpression). Finally, whether HELLS acts independently of other mechanosensitive factors such as YAP warrants further investigation, for instance via YAP knockdown under force application.

In conclusion, our study suggests HELLS as a novel compressive force-responsive gene. The downregulation of HELLS may contribute to OTM through dual mechanisms: impairing osteogenic differentiation and potentiating osteoclastogenic signaling.

## 4. Materials and Methods

### 4.1. Animals and Orthodontic Tooth Movement Model Establishment

Seven healthy male C57BL/6 mice (6–8 weeks old, 20–25 g) were obtained from Vital River Laboratory Animal Technology (Beijing, China). Mice were housed under controlled temperature conditions with a 12 h light/dark cycle and were provided a softened diet to minimize discomfort during orthodontic force loading.

The OTM model was generated as described previously, with minor modifications [27]. Mice were anesthetized via intraperitoneal injection of 1% sodium pentobarbital. A nickel–titanium tension spring (0.12 mm wire diameter) was attached to apply a calibrated 50 g mesial force to the right maxillary first molar, while the contralateral molar served as an internal control. The spring was secured using a stainless steel ligature wire passed through the interproximal space, and the anterior end was anchored to the maxillary incisors. A constant force of 50 g was precisely calibrated and monitored using a dynamometer to ensure consistent mesial displacement of the first molar.

### 4.2. Sample Collection and Histological Processing

Mice were euthanized 14 days after force application. The maxillae, including the first molars and surrounding periodontal tissues, were harvested and immediately fixed in 4% paraformaldehyde at 4 °C for 24 h. Subsequently, the specimens were decalcified in 10% ethylenediaminetetraacetic acid (EDTA, pH 7.4) for approximately 4 weeks, with the decalcification solution refreshed every 3 days. Following complete decalcification, the tissues were dehydrated through a graded ethanol series, embedded in paraffin, and sectioned into 5-μm-thick sagittal serial slices for subsequent histological and immunofluorescence staining.

### 4.3. Immunofluorescence Analysis

The slices obtained from the maxillary specimens were blocked with 5% normal goat serum (NGS) in PBS for 1 h at room temperature, and then incubated overnight at 4 °C with rabbit anti-HELLS primary antibody (11955-1-AP, ProteinTech, Rosemont, IL, USA) diluted 1:200 in PBS containing 1% BSA. For cell experiments, hPDLSCs seeded on coverslips were transfected with siHELLS or siNC. Coverslips were fixed, permeabilized with 0.2% Triton X-100 and blocked as above, and then incubated overnight at 4 °C with primary antibodies against HELLS (11955-1-AP, ProteinTech, 1:200 in PBS/1% BSA) and phospho-γH2AX (GB111841, Servicebio, Wuhan, China, 1:200 in PBS/1% BSA). After three washes with PBS, samples were incubated with Alexa Fluor 488 conjugated anti rabbit IgG (A32731, Thermo Fisher, Waltham, MA, USA, 1:500) for 1 h at room temperature in the dark. Nuclei were counterstained with DAPI (1 μg/mL). Images were captured using confocal microscopy. HELLS fluorescence intensities were quantified using ImageJ 1.52a and compared between force-loaded tissues and contralateral unloaded controls.

### 4.4. Cells and Treatment

Human PDLSCs (hPDLSCs) were provided by the ORAL STEM CELL BANK of Beijing Tason Biotech Co., Ltd. (Beijing, China) (http://www.kqgxb.com). Cells were cultured in α-MEM supplemented with 10% fetal bovine serum and 1% penicillin/streptomycin. hPDLSCs from passages 4 to 6 were utilized for all experiments to ensure phenotypic stability. The murine macrophage cell line RAW 264.7 was obtained from Servicebio (Wuhan, China). Cells were cultured in Dulbecco’s modified Eagle’s medium (DMEM) supplemented with 10% FBS and 1% penicillin/streptomycin. The cells were maintained in a humidified incubator at 37 °C with 5% CO_2_.

Static compressive force was applied on hPDLSCs as previously described [10,27,28]. Briefly, hPDLSCs were seeded into 6-well plates and cultured until they reached 80% confluence. Then, a sterile cover glass and a glass cylinder filled with sterilized stainless steel granules were placed directly onto the cell monolayer. The total weight was adjusted to achieve the desired compressive stress (1.0 or 1.5 g/cm^2^), calculated as total weight (g) divided by the contact area (cm^2^) of the bottle bottom. Control cells were cultured under identical conditions without the cover glass, glass cylinder and beads. For time-course analysis, cells were exposed to 1.0 g/cm^2^ for 6 and 12 h. For magnitude-dependent analysis, cells were subjected to 1.0 or 1.5 g/cm^2^ for 6 h. hPDLSCs were treated with BAY 11-7082 (5 μM, MCE) or RANKL neutralizing antibody (15 ng/mL, Thermo, MA5-29614) for 12 h and then subjected to force of 1.0 g/cm^2^ for 12 h.

### 4.5. Small-Interfering RNA (siRNA) Transfection

Small-interfering RNAs (siRNAs) against HELLS and a scrambled control siRNA was chemically synthesized (GenePharma, Shanghai, China). Cells were transfected with 50 nm siRNA oligonucleotides for 72 h using lipofectamine RNAiMAX (Invitrogen, Waltham, MA, USA). The siRNA sequences were as follows: siHELLS, GUUGUUUAUCGCCUUGUUA, siE2F1: GACGUGUCAGGACCUUCGU, and siNC, UUCUCCGAACGUGUCACGU.

### 4.6. RNA Isolation and Quantitative Reverse-Transcription Polymerase-Chain Reaction (qRT-PCR) Analyses

Total RNA was extracted using TRIzol reagent (Invitrogen, Waltham, MA, USA) according to the manufacturer’s instructions. RNAs were reverse-transcribed into cDNA. The messenger RNA levels of indicated genes were quantified via quantitative polymerase chain reaction (qPCR) and normalized to GAPDH. The 2^−ΔΔCt^ method was employed to determine the relative fold changes in gene expression. The primer sequences for each gene are listed in Table 1.

### 4.7. Osteogenic Differentiation Induction, Alizarin Red Staining (ARS), and Alkaline Phosphatase Activity Assay

Cells were maintained in osteogenic differentiation medium supplemented with β-glycerophosphate, ascorbic acid, and dexamethasone. After 14 days, early osteogenic differentiation was assessed by ARS and ALP activity assays using commercial assay kits (Beyotime, Beijing, China).

### 4.8. Transcriptome Sequencing (RNA-Seq) and Bioinformatics Analysis

hPDLSCs were transfected with HELLS-targeted siRNA or negative control siRNA (siNC) for 48 h. Total RNA was extracted using the TRIzol reagent according to the manufacturer’s instructions (Invitrogen, Waltham, MA, USA). RNA quality and integrity were assessed, and qualified samples were used for library construction. High-throughput paired-end sequencing was performed on an Illumina platform (HiSeq, Novogene, Beijing, China).

For the bioinformatics analysis, raw reads were processed for quality control, followed by read mapping to the reference genome and quantification of gene expression levels. Differentially expressed genes (DEGs) were identified based on established thresholds (|fold change| ≥ 1.5 and *p* < 0.05). Functional enrichment was then evaluated via Gene Ontology (GO) and Kyoto Encyclopedia of Genes and Genomes (KEGG) analyses to elucidate the transcriptional landscape and regulatory networks modulated by HELLS knockdown in hPDLSCs.

### 4.9. Western Blot Analysis

Western blot analysis was performed as previously described [29]. The following antibodies were used: HELLS (ProteinTech, 87169-1-RR, 1:2000), E2F1 (12171-1-AP, 1:1000), YAP (CST, #4912, 1:1000), GAPDH (CST, #2118, 1:2000). Primary antibodies were diluted in 5% BSA/TBST. HRP-conjugated secondary antibody (anti-rabbit, 1:5000, ProteinTech) was used.

### 4.10. Preparation of Conditioned Medium, Osteoclast Differentiation Induction, and Tartrate-Resistant Acid Phosphatase (TRAP) Staining

PDLSCs were seeded into six-well plates and transfected with HELLS siRNA or control. Supernatants were mixed with fresh culture medium at a 1:1 ratio and added to RAW264.7 cells. Osteoclast differentiation was then induced in the presence of RANKL (25 ng/mL) and MCSF (20 ng/mL). The cells were subsequently collected for qRT-PCR analysis and TRAP staining using a TRAP staining Kit (Solarbio, Beijing, China).

### 4.11. Statistical Analyses

For statistical analyses, Student’s *t*-tests or one-way analysis of variance (ANOVA), as indicated in the relevant figure legends, were performed using GraphPad Prism 9. All data are expressed as mean ± standard deviation. Statistical significance was considered at *p* < 0.05.

## Figures and Tables

**Table 1:** Primer Sequences for Quantitative Reverse-Transcription Polymerase Chain Reaction (qRT-PCR).

Target	Forward Primer	Reverse Primer
*GAPDH*	AATTCCATGGCACCGTCAAG	ATCGCCCCACTTGATTTTGG
*HELLS*	TGAACAACTGGACACTGCTG	AAGACATGCGAGCCTTTTCC
*RANKL*	TGGAGAGGAAATCAGCATCGAG	GCCCCAAAGTATGTTGCATCC
*OPG*	TGGACATGCTAACCTCACCTTC	ATTCGCCACAAACTGAGCAG
*E2F1*	TGAGGAGTTCATCAGCCTTTCC	TCCCCAAAGTCACAGTCGAAG
*Trap* (mouse)	ACTTGCGACCATTGTTAGCC	AGAGGGATCCATGAAGTTGC
*Ck* (mouse)	AAGGCAGCTAAATGCAGAGG	TTGCATCGATGGACACAGAG
*Oscar* (mouse)	TCAACGTGACCTTGACTTGC	AAGAACTCAGCCAGCTCAAC
*Gapdh* (mouse)	AACGACCCCTTCATTGACCTC	ACTGTGCCGTTGAATTTGCC

## Data Availability

The datasets and materials generated in this study are available from the corresponding author upon request.

## Supplementary Materials

The following supporting information can be downloaded at: https://www.mdpi.com/article/10.3390/ijms27104540/s1.
