# Supplementary material for: HELLS Reduction Contributes to Compressive Force-Induced Functional Changes in PDLSCs
Source: Int J Mol Sci. 2026 May 19;27(10):4540. doi: 10.3390/ijms27104540 (PMC13207725; doi:10.3390/ijms27104540)
Supplement: Supplementary file 1 [file ijms-27-04540-s001.zip › ijms-4214320-supplementary.pdf]

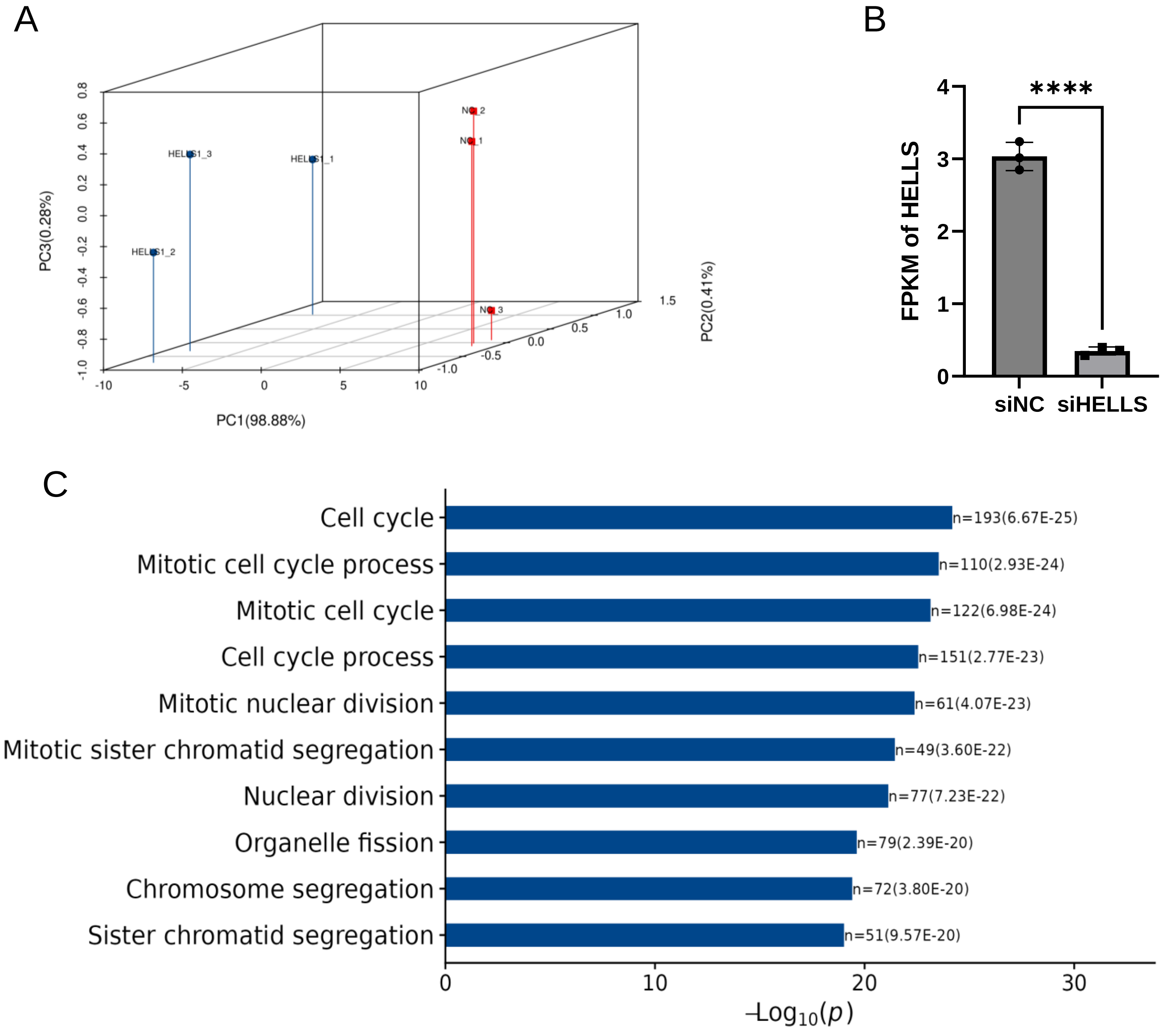

Supplemental Figure S1. RNA-seq analysis of hPDLSCs following HELLS knockdown. (A) Principal component analysis of RNA-seq data for hPDLSCs transfected with HELLS-specific siRNA (siHELLS) or a negative control siRNA. (B) HELLS expression levels (FPKM, Fragments Per Kilobase of transcript per Million mapped reads) from the RNA-seq dataset (n = 3; Student's t-test, ****P < 0.0001). (C) Top 10 terms of Gene Ontology (GO) enrichment analysis of DEGs.

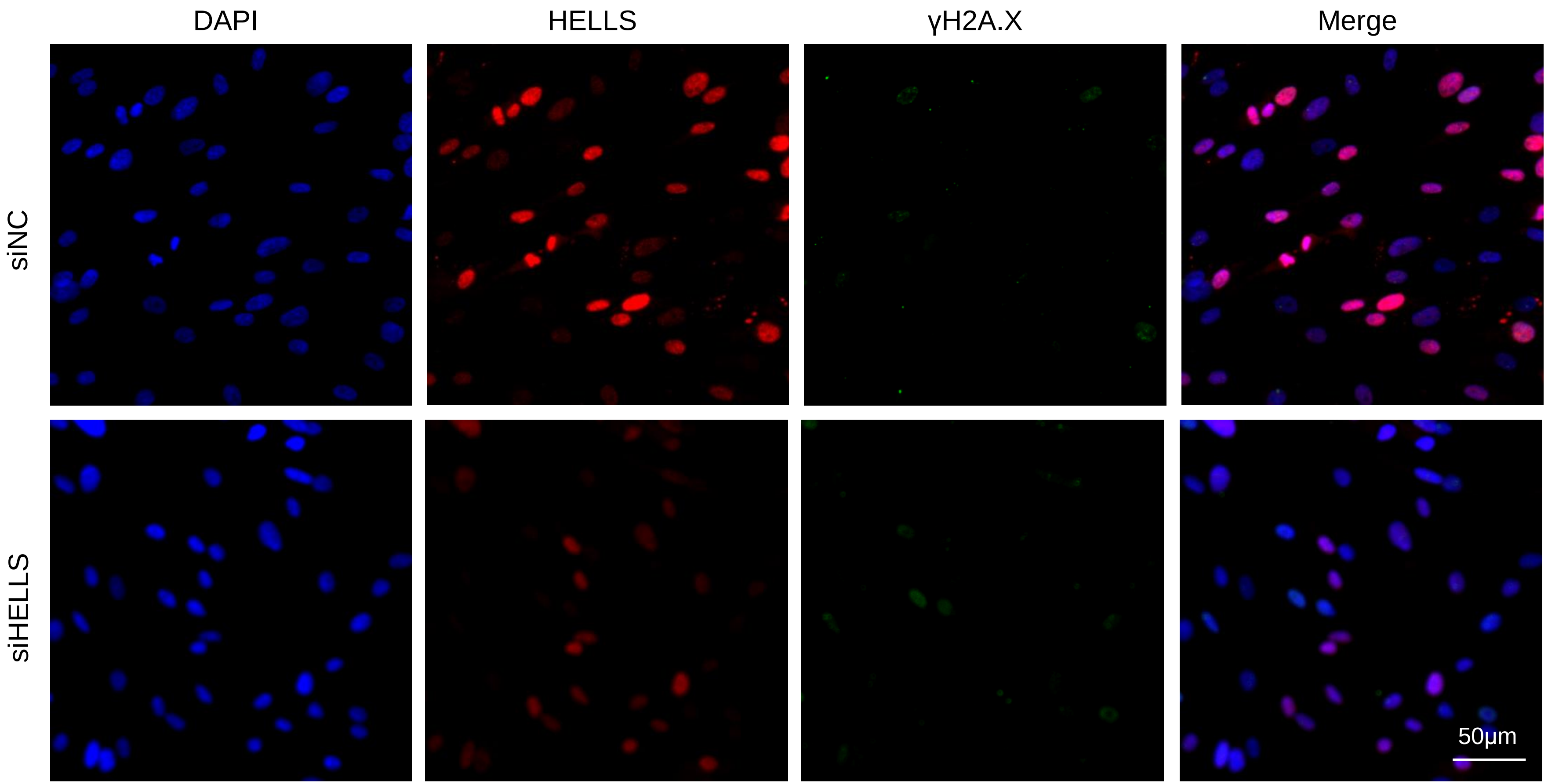

Supplemental Figure S2. Immunofluorescent analysis of γH2A.X in hPDLSCs following HELLS knockdown. Cells were transfected with siRNA targeting HELLS (siHELLS) or a negative control (siNC), and then stained for HELLS and phosphorylated γH2A.X (γH2A.X).
