# Supplementary figures and images for: HELLS Reduction Contributes to Compressive Force-Induced Functional Changes in PDLSCs
Source: Int J Mol Sci. 2026 May 19;27(10):4540. doi: 10.3390/ijms27104540 (PMC13207725; doi:10.3390/ijms27104540)

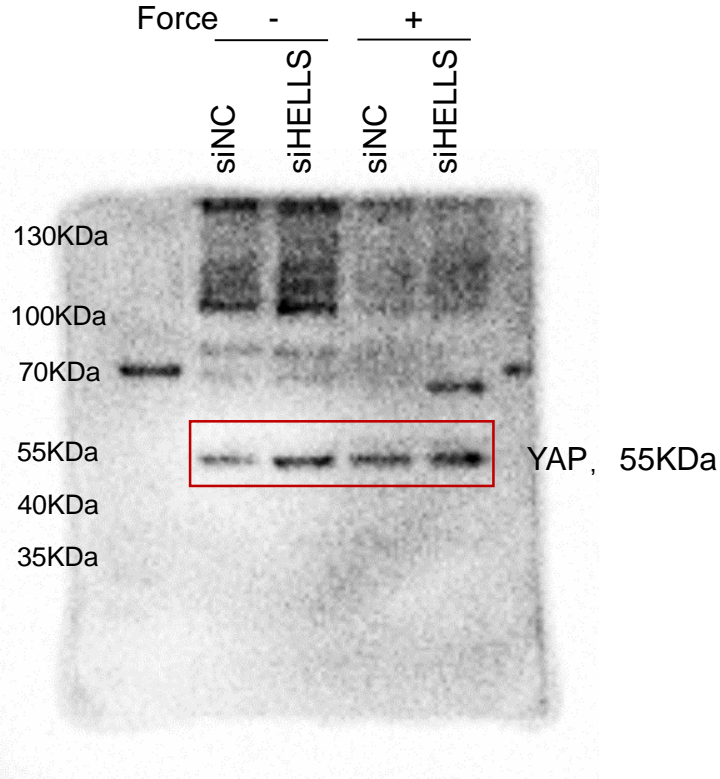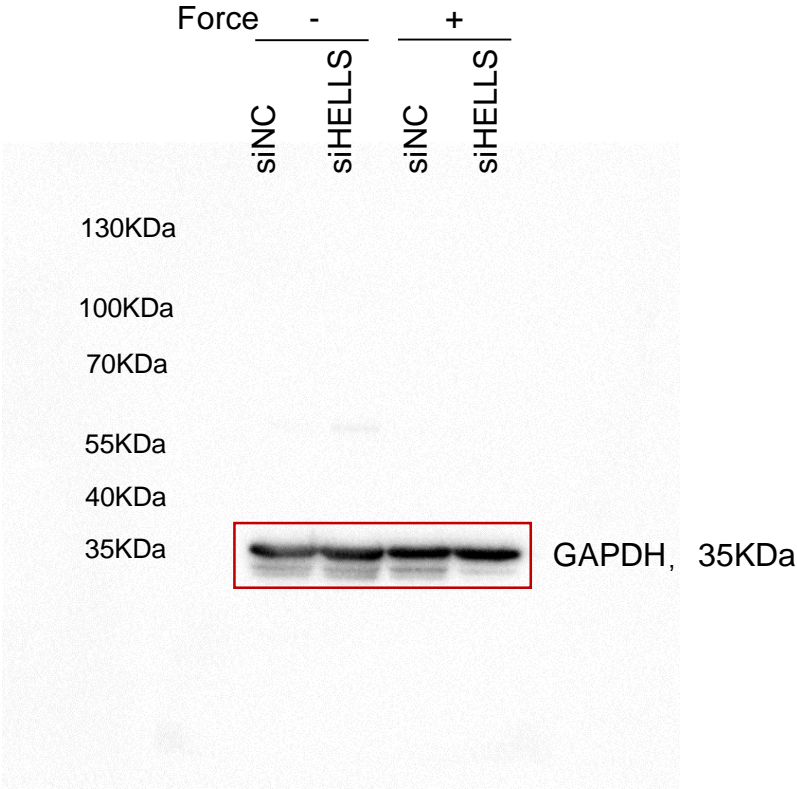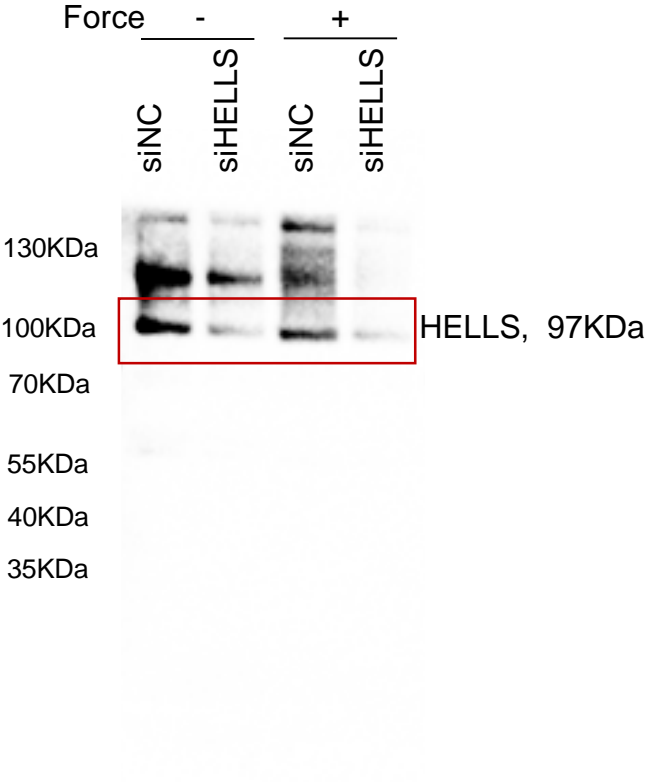

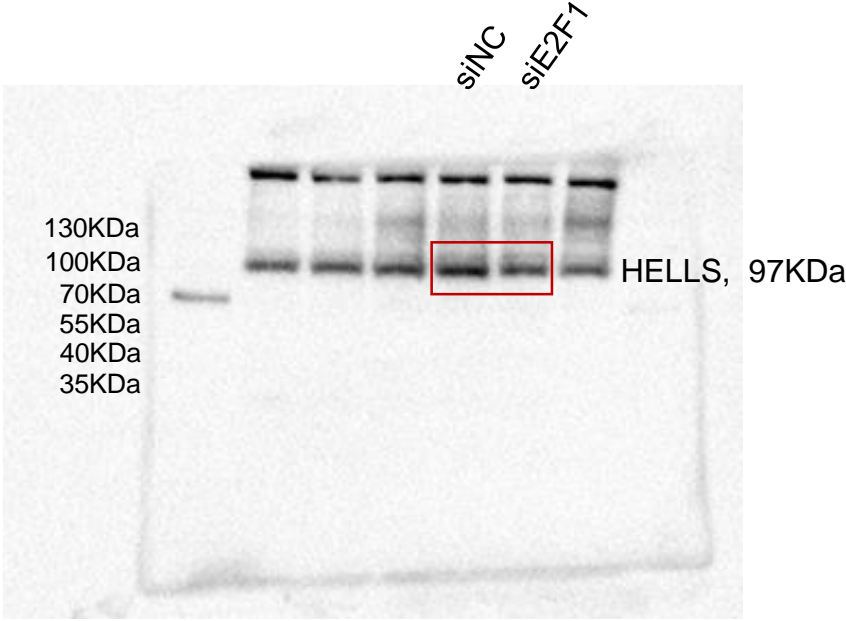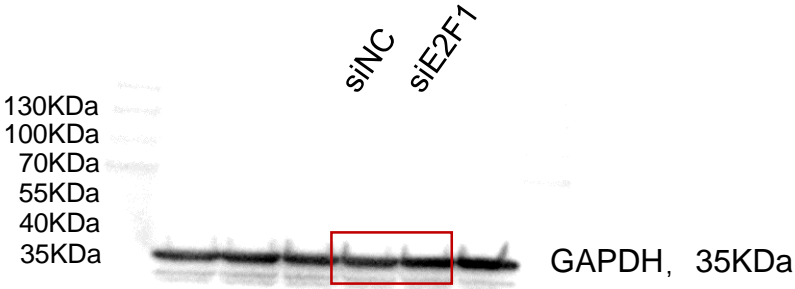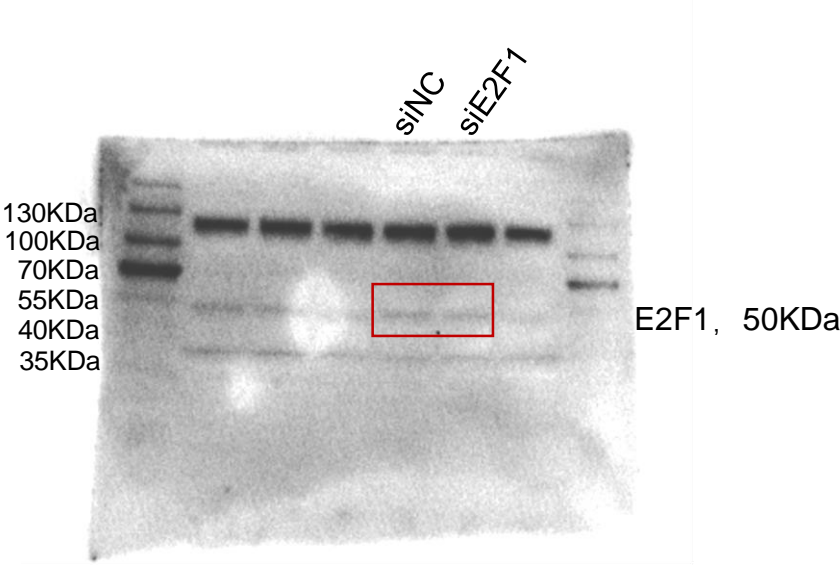

Supplement: Supplementary file 1 [file ijms-27-04540-s001.zip › Supplementary Figures-Source data for WB.pdf]
